# Service Priority and Standard Performance of Community Mental Health Centers in South Korea: A Delphi Approach

**DOI:** 10.4306/pi.2009.6.2.59

**Published:** 2009-06-30

**Authors:** Myung-Soo Lee, Maanse Hoe, Tae-Yeon Hwang, Young Moon Lee

**Affiliations:** 1 WHO Collaborating Center for Psychosocial Rehabilitation, Yongin Mental Hospital, Yongin, Korea.; 2 Seoul Mental Health Center, Seoul, Korea.; 3 School of Social Work, University of Southern California, Los Angeles, USA.; 4 Ajou Institute of Mental Health, Department of Medical Humanities and Social Medicine, Suwon, Korea.

**Keywords:** Community mental health center, Service priority, Delphi survey

## Abstract

**Objective:**

The role of community mental health centers (CMHCs) in Korea is quite different than that of these centers in Western countries due to nation-specific health care system characteristics. For example, CMHCs of Korea are expected to provide services for serious mental illness in addition to other services in response to community needs, such as internet addiction of adolescents. Consequently, it is important to determine service priorities of CMHCs and to define standard service performances in order to maximize their effectiveness with limited resources. The present study aimed to generate expert consensus on service priorities and to identify standard service performances of CMHCs in South Korea.

**Methods:**

Forty-five mental health professionals participated as experts in a Delphi survey. We made a survey questionnaire based on Korean and international data and guidelines of some countries such as the UK and Australia. Experts answered the first and second round questionnaires and their answers were analyzed using frequency analysis.

**Results:**

For the question about future directions of CMHCs, twenty-two experts (49%) answered that the growth of services for serious mental illness should be preferred to other areas. The service for chronic mental illness was thought to be the most important service area (27.1%) and, early psychosis (10.5%) is included, the services for serious mental illness should be regarded as the most important service area of Korean CMHCs. It is followed by child and adolescent services (13.2%) and mental health promotion services (10.8%). The relative importance of service performances on each service domain were given by answers of experts.

**Conclusion:**

CMHCs in Korea should focus their priority on the management of serious mental illness. Service standardization by the relative importance of service performances on each service domain is needed.

## Introduction

For the past 50 years, Western countries have been deinstitutionalizing their mental health system, leading to a greater focus in community mental health care. There are some criticisms about the quality of care, its effectiveness and limitations, such as receiving no treatment, becoming homeless and/or ending up in prison.^1^-^3^ Regardless of those limitations, the community-based mental health system is no longer the substitute for long-term institutionalization, but rather is now the main stream center of care. This reform process was an inclusive one in which mental health professionals, budget and locus of care were comprehensively shifted from institutions to communities.^4^-^7^ Despite sharing common experiences with Western countries, however, the deinstitutionalization process and the current community service systems differ from country to country, depending on many factors such as specific traditions, socio-economic situations and funding arrangements.^8^-^10^

Korea has taken a different approach in the deinstitutionalization and downsizing of hospitals. The number of psychiatric beds continues to increase (1.39 beds per 1,000 population, as of 2007) and patients' length of stay in psychiatric hospitals has not decreased (on average 155 days in 2007),^11^ while simultaneously increasing the infrastructure for community services. As of 2008, 148 out of 232 districts (64%) have community mental health centers (CMHCs). There are also facilities for social rehabilitation, such as day care centers, residential services and vocational rehabilitation services and these facilities continue to increase. These differences might be due to several unique characteristics of Korea. First, the high proportion of the private sector as a service provider (inpatient and outpatient treatments) hinders the Korean government from establishing policy to control psychiatric beds. Second, because the budget system for hospital and community services is fragmented, the increase and decrease of hospital services does not directly affect the variations of community services.

Western countries, especially Western Europe and Australia, have set up their community mental health system based on the concept of sectorization. For example, Ireland has created separate substance misuse and mental health services for adults, children and adolescents and older adults for every 300,000 people.^12^ Australia also set up a catchment area for every 200,000 to 300,000 people with child and adolescent, adult mental health, older adults, and statewide specialized mental health services.^13^ On the contrary, Korean CMHCs (as representative for community services) have been required to respond to all mental health needs, which cannot be done by them due to shortage of resources. The Korean government assigns low priority to developing community based mental health services, causing CMHCs to face this shortage of resources. In addition, the Korean government does not have the intention to replace long-term hospital cares with community-based cares in order to downsize health cost and to increase quality of services because long-term hospital care is relatively inexpensive. This is because the medical fee schedule for inpatient services is set very low, especially for Korean Medicare patients: the government pays only 1,000USD/month for Medicare inpatient services regardless of the amount of services provided. In this situation, Korean CMHCs must develop an efficient service system which calls for standard service guidelines.

The World Health Organization published a mental health service guidance package that consists of key recommendations for service performances.^14^ Many Western countries also have their own service guidelines which include key performance indicators and identification of key categories of community services.^15^-^17^ Nonetheless, for the past 10 years the CMHCs in Korea have focused mainly on case management for patients with persistent and severe mental illness. In a survey conducted in 2006,^18^ the CMHCs placed a priority on services for the seriously mentally ill, with an average of 45% of the total services provided. Recently, CMHCs have been required to provide diverse services, such as internet addiction counseling, in response to community needs. Consequently, deciding the CMHC service priorities are important in order for CMHCs to maximize service effectiveness, while avoiding confusion concerning service goals and future directions at the individual CMHC level. However, the Korean government has not yet established well standardized performance guidelines. Setting up standardization could be very important, especially for the Korean CMHCs which have to cover various population groups with limited resources.

The present study aimed to identify service categories in CMHCs and to generate expert consensus on CMHC service priorities of standard performance on the identified service categories in S. Korea. Expert consensus on the CMHC service priority and on the standard performance will be informative in developing a more advanced community-based mental health service system that will make it easier for people with mental health problems to receive adequate care. The expert consensus using a Delphi survey technique will facilitate discussion on future directions for the Korean community-based mental health service system by diverse mental health professionals.

## Methods

### The delphi process

The present study used a Delphi survey technique, an effective method to produce an expert consensus.^19^-^21^ The Delphi survey technique is advantageous in reaching group consensus about goals, processes, and future directions by means of experts' knowledge and judgment. This technique is also useful to facilitate communications between government and experts for decision-making.^22^ The survey was conducted during 3 months, from February to April in 2007.

### Expert panel

Forty-five mental health professionals who had experiences in community mental health systems were sampled. Region, position and professional background of the participants were considered. The participants consisted of 19 psychiatrists (42.2%), 7 psychiatric nurses (15.5%), 9 social workers (20%) and 10 government officials (22.2%). All the panels have more than 5 years experiences as a director (37.7%) or team manager (20%) of CMHC or as a member of community mental health service supporting committee (22.2%) or government officer (20%). The response rate was 100% on first and second round survey.

### Development of the questionnaire

Delphi survey questionnaire were developed using findings from a nationwide survey regarding staff, budget and task analysis of CMHC,^18^ the mental health service guideline,^23^ an annual report of the Central Mental Health Supporting committee^11^ and the Standardized Mental Health Service Guidelines of Australia^24^ and U.K.^25^,^26^ The Delphi survey questionnaire asked participants to provide their opinions on three issues: the role and future direction of CMHCs, the current and future priority of service domains and sub-domains and the relative importance of service performances. There were four service domains: 1) specialized mental health service for serious mental illness management, 2) primary mental health services for high prevalence mental health problems (e.g., depression, suicide, alcohol addiction, geriatric metal health), 3) prevention and promotion and 4) planning and research. The domains and sub-domains are presented in Table 1. We also listed service performances and provided their operational definitions for the expert participants (Table 2). The service performances were counseling, clinical counseling, group program, education, crisis management, pharmacological therapy, screening, community networking, public relations, campaign, and community assessment.

### First round

The first round was designed to collect participants' opinions on three questions concerning CMHCs. The first question intended to establish consensus on the role and future direction of CMHCs taking into consideration the current social situation - the necessity of strengthening service systems for chronic and serious mental illness while meeting the increasing need of the community for various mental health issues. In the second question, participants were asked to provide their priority proportion on service domains and sub-domains for the present and the future. The third question was about the relative importance of service performances. The level of relative importance was measured from zero to five. The score of five indicated the most important performance in CMHCs while zero refers to a service which CMHCs do not need to provide. We intended to report the consensus about what service performances would be selected as standard performances which should be applied to overall mental health service spectrum.

### Second round with additional questions

We sent participants the results of the first-round survey and second-round questionnaire through e-mail. Their responses were collected through fax and e-mail. The second-round questionnaire included all questions from the first round and additional questions in order to elaborate participants' opinions on service priority further. The additional questions consisted of the following issues: services for alcohol addiction and for homeless people, questions about group programs and pharmacological therapy at the CMHCs.

## Results

### General directions for community mental health centers

The major findings for the first question are as follows: 22 participants (49%) felt that the growth of services for serious mental illness needed to be prioritized instead of other areas, while 18 participants (40%) felt that the growth of other service areas needed to be prioritized rather than services for serious mental illness. Only 2 participants (4.5%) felt that the services for serious mental illness should be reduced (6%) and 3 (6.7%) thought that it should be converted completely into mental health promotion programs. 8 participants (18%) changed their responses during the second round.

### Service priority of community mental health centers

**FIGURE 1:**
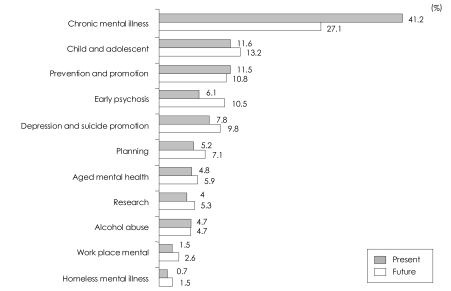
Service priority changes and ranking on a future basis.

Participants were asked to weigh twelve sub-domains for their relative importance to CMHCs for providing their services. Table 1 showed participants' opinions on service priority of CMHCs. Chronic mental illness was ranked as the most important, services for child and adolescents was ranked as the second most important, followed by prevention and promotion, services for early psychosis, depression and suicide prevention. Alcohol addiction and homeless mental illness were ranked the least important of the services. As seen in Table 1 and Figure 1, participants indicated that service allocation for the management of chronic mental illness could be reduced by 14%, but felt that services for early psychosis, including first-episode psychosis, should be increased by 4.4%. Consequently, services for serious mental illness were thought to be the most important service in CMHCs.

### Standard performance of community mental health centers

Table 2 shows the relative importance of each service performance in CMHCs. This result suggests the role of CMHCs in the whole spectrum of the community mental health service system.

### Issue of prescription availability at community mental health centers and other results

At first- and second-round, pharmacological treatment scored a median value of 0. For additional questions in the second round, 67.4% of psychiatrists and 80% of nonpsychiatrist experts indicated that CMHCs could provide temporary pharmacological treatment in restrictive cases. There was no statistical difference between the two groups (p>0.05). Substance abuse management and services for homeless mentally ill were of relatively low priority in this study. Participants answered additional questions in the second round of the survey which showed that it is necessary for CMHCs to do basic counseling, provide information and refer to treatment for substance abusers and homeless mentally ill.

In the first round of the survey, the importance of providing group program as a service performance scored 3 across three sub-domains; chronic mental illness, child and adolescent, as well as depression and suicide prevention (Table 2). The additional questions revealed participants' opinion that CMHCs' functions could differ depending on whether social rehabilitation facilities exist in their catchment areas. That means CMHC can meet consumer's needs in collaboration with other social rehabilitation facilities if those facilities exist.

## Discussion

CMHCs in Korea have rapidly increased over the past 14 years and have played a key role in providing mental health services to the community. The recent demands by the Korean government for CMHCs to provide new, community-based services, such as school mental health promotion programs, suicide prevention, alcohol and internet addiction, have caused confusion and additional burdens for mental health practitioners due to having to prepare new services with limited resources. Mental health practitioners suffer from having high caseloads in serving clients with serious mental illness. Thus, there is a need for standard service guideline for CMHCs to build efficient community-based mental health services. Findings from the present study have important implications for developing these guidelines.

The forty-five mental health experts who participated in our Delphi survey agreed that the Korean CMHCs should focus primarily on services for the seriously mentally ill. This result was consistent with the emphasis on the accountability of CMHC for serious mental illness in the newly amended Mental Health Act in 2008. For example, the adoption of community treatment order.^27^ However, the experts also agreed with the necessity to respond to new community needs and pointed out that optimalization of input will be a prerequisite for establishing more comprehensive services. This result led us to conclude that CMHCs in Korea are required to provide comprehensive services which cover both traditional mental health services and new services. Accordingly, we suggest two types of service performance for CMHCs: Primary Mental Health Services (PMHS) and Specialized Mental Health Services (SMHS).

PMHS refers to psychological counseling, screening for mental health problems, information providing services and a referral to psychiatric treatments for all population groups. SMHS refers to intensive mental health services, such as intensive case management for serious mental illness, as well as for chronic or early psychosis. SMHS will also include crisis intervention services for people at high risk for suicide and for acutely psychotic patients. These services can be delivered by the CMHCs, either solely and directly or through community networking. Activities such as education and public relations can be commonly performed both in PMHS and SMHS.

The role of CMHC varies from country to country and the role of Korean CMHC is different from that of Western countries. The Korean system consists of a large number of private psychiatrists who run their own community clinics or who work in private hospitals (5.5 psychiatrists per 100,000 populations). This has influenced government to set up CMHCs without pharmacological treatment capability. It is true that policy makers and program planners need to consider the nation-specific characteristics of the health care system when developing public mental health service delivery systems, but they should also consider the limitations of current health care system. For example, the fact that involuntary admission rates by a family member was 88.2% of the total admissions in Korea^11^ suggests that CMHCs have not developed effective service systems for crisis interventions and that CMHCs need pharmacological treatment capability. Therefore, the government should consideration allowing CMHCs to provide restricted pharmacological therapy for patients who are neglected in the community and who suffer from a mental health crisis without adequate support. The debate about pharmacological treatment at the CMHC has been repeatedly discussed among mental health professionals in Korea. To address this, we added additional questions about pharmacological therapy at CMHCs to the second round survey. Approximately 70% of the experts agreed that there was need for temporary medication treatment for low income patients with no social support, patients who did not have an understanding of the illness and homeless patients who could not afford medications.

As consumer-centered comprehensive services are demanded, CMHCs need to cover various rehabilitative services, such as vocational rehabilitation and social skill training. Our findings indicate that the types of service programs offered in CMHCs would be related to the presence of social rehabilitation facilities within the same catchment area. Our expert participants did not agree that group programs should be a key performance activity at CMHCs. This opinion reflects the fact that social rehabilitation facilities neighboring CMHCs provide various group programs and thus group programs do not take a priority in CMHCs. This result is consistent with our finding that CMHCs' function could differ depending on whether social rehabilitation facilities exist in their catchment areas. However, a survey conducted in 2006^18^ reported that 42.9% of CMHCs did not have social rehabilitation facilities within their catchment area. Thus, many CMHC indeed face demands to provide psychosocial rehabilitative programs. This reality was evident when participants emphasized that well designed and specialized group programs could be provided in CMHCs regardless of the existence of social rehabilitation in their catchment area.

Our findings from the Delphi survey with forty-five mental health experts are informative and will help in deciding the future directions of CMHCs, the priority of services and standard service performances. As a next step, we need to develop standard service guidelines for CMHCs to do for the better outcomes. This study was limited to exploring these issue and to making a consensus about the priority of services and service performances. It is necessary to establish evidence on each service performance, which could help Korean CMHCs to provide the best, evidence-based practices for the community.

## Figures and Tables

**TABLE 1:**
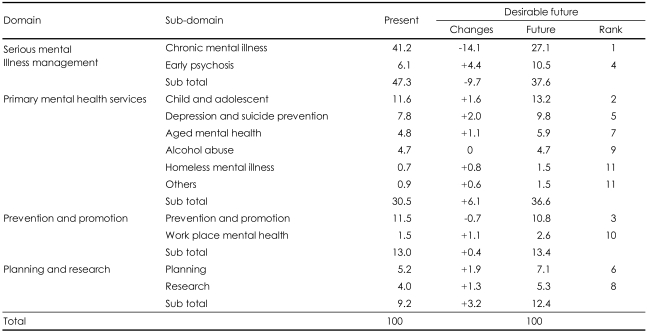
Service priority changes between present and the future (%)

**TABLE 2:**
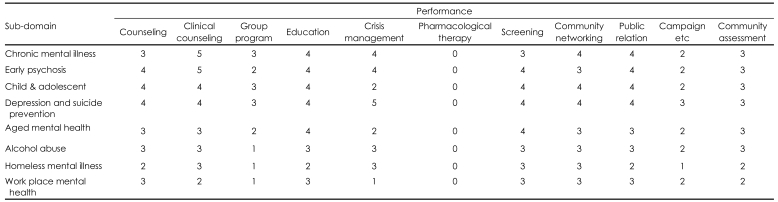
Relative importance of service performances in community mental health center (median number)

5: most important service performances, 4: important service performances. Operational definitions of service performances. Counseling: providing information for mental health problems, Clinical counseling: therapeutic intervention in the process of case management, Group program: group programs for psychosocial rehabilitation, vocational rehabilitation and so on, Education: psychoeducation for patients and carers by individual or group level, Crisis management: activities of evaluation and intervention for psychiatric crisis, such as emergency outreach, intervention for acute admission and so on, Community networking: all the activities for facilitating early detection, intervention and rehabilitation in collaboration with the community agencies, Screening: individual-based psychological assessment, Community assessment: population-based epidemiological assessment, needs assessment and so on
